# Wide-Sense Nonblocking Converting-Converting Networks with Multirate Connections

**DOI:** 10.3390/s22166217

**Published:** 2022-08-18

**Authors:** Wojciech Kabaciński, Remigiusz Rajewski

**Affiliations:** Institute of Communication and Computer Networks, Poznan University of Technology, Polanka 3, 60-965 Poznan, Poland

**Keywords:** multirate networks, 2-stage networks, wide-sense nonblocking (WNB), elastic optical networks

## Abstract

In this paper, we consider a two-stage converting-converting (CC) switching network. This structure can be used, for instance, in switches of elastic optical networks (EONs) or in time-division switches. We propose a new routing algorithm based on fixed slot assignment in interstage links. This algorithm, called Fixed Input–interstage Slot Assignment (FISA), reduces the switching network complexity compared to the rearrangeable (RNB) switching networks of the same structure. We derive the wide-sense nonblocking (WNB) conditions for the switching network controlled by this algorithm. The obtained WNB conditions are the same as those of the RNB, but the switching network does not need troublesome and time-consuming rearrangements. When implementing the proposed switching network structure, we can also reduce the number of tunable full-range spectrum converters and replace part of them with fixed spectrum converters, or even use space switches in the first stage. This is especially important when this architecture is applied in EONs.

## 1. Introduction

The Internet of Things (IoT) is a network of interconnected objects equipped with sensors and other devices that exchange data. They include everyday household objects and complex tools used in various technological and production processes. In many applications, data transmission between devices must be error-free, reliable, and slightly delayed, that is, meet specific quality parameters, Quality of Service (QoS). Many factors influence the parameters obtained, for example, the quality of the sensors, technical parameters of the measuring devices, the quality of access connections and the structure of the network itself. In most studies, performance evaluation is carried out using simulation models. In these models, various network topologies are built, multiple transmission link parameters are adopted, or different protocols for information exchange are used. Most studies assume that data transfer in the network nodes itself is lossless. It is especially true for router switching networks. For this condition to be met, these switching networks must be properly constructed and use routing algorithms that match the topology. Ensuring the lossless nature of the switching fabric is associated with its cost. Still, the cost is also affected by the type of network in which the node is used, transmission speed on input and output links, or types of connection used in the node (single-rate or multi-rate, unicast or multicast, etc.).

Today, networks serve connections that use a wide range of bandwidths. The available bandwidth in the transmission media is often divided into small portions called slots and assigned to connections on request. In time-division switching networks, we have time slots [1], while in optical networks, we have frequency slots [2]. Slots are also called channels. In general, *m* slots can be assigned to a connection, where m≤n and *n* is the number of all channels available on a link (that is, the link’s capacity). This connection is denoted as an *m*-slot (*m*-channel) connection, has to use slots in one link, and in some applications, has to use successive slots. Examples are synchronous optical packet networks that serve variable-length packets (such a packet is transmitted through several successive time slots [3] or connections in flexible optical networks [2,4,5]). Flexible (or elastic) optical networks are the new paradigm proposed for optical transport networks to use available bandwidth more efficiently. The International Telecommunication Union defined the size of Frequency Slot Unit (FSU) as 12.5 GHz [2], and depending on the required transmission rates, *m* adjacent FSUs can be assigned to an optical channel (connection).

Connections in communication networks are routed through network nodes. Technological constraints limit the capacity of integrated switches. Thus, many of these switches are interconnected in large network nodes and form a multistage switching network. One of the most commonly known structures is the three-stage Clos switching network [6] and its different variants [7,8,9,10,11].

Strict-sense Nonblocking (SNB), Rearrangeable Nonblocking (RNB), RePackable Nonblocking (RPNB), and Wide-sense Nonblocking (WNB) (also called combinatorial properties) of switching networks determine the conditions under which a new connection between a free input–output port pair can be set up (that is, there is no blocking state). Definitions of these properties are provided, for example, in [1,12,13]. The nonblocking networks differ in the way blocking states are omitted. In SNB networks, blocking states never exist for any connection and for any routing algorithm used. In RNB and RPNB networks, blocking states can always be omitted by rearrangements, that is, by moving some existing connections to other connecting paths. The difference is when these rearrangements are executed. When they are invoked when a newly arrived connection is blocked, we say that the network is RNB. In RPNB networks, the repacking algorithm is used to rearrange the state when one of the existing connections is terminated to realize any future connection without blocking. Finally, WNB networks allow one to omit blocking states without rearrangements, provided that an appropriate routing algorithm is used. In nonblocking networks, the probability of blocking caused by the inaccessibility of the internal route is reduced to zero. The blocking state can be omitted when evaluating the performance of the switch or network. Therefore, one of the parameters used to characterize the switching node is the nonblocking property of its switching networks. A short survey of the combinatorial properties of various switching networks is provided in Section 2.

If there is a blocking state in an optical switching node, it means that this node cannot set up some optical connection. Therefore, this connection needs to be retransmitted later through this same switching node or should be directed through a different network’s nodes (if possible at all). In both cases, additional time is needed. Moreover, traffic in optical fiber is also increasing; we need to send this exact connection many times. For the end user, it means that its perception can feel the lower quality of some streaming multimedia; downloading some data are more time-consuming, and so on. For the operator’s perception, it means that no retransmission is needed and the operator (or network provider) can serve more services in the same amount of time. Generally, the node is the heart of future optical networks, especially in the core network. Without a very efficient node, we cannot think about other parameters, especially QoS or Quality of Experience (QoE). Therefore, we consider in our article how to construct a switching node and how to route connections to omit blocking states. The probability of blocking in the switching network is the most critical quality parameter for assessing a switch node (such as, for example, a router or switch). In a nonblocking switching network, this probability is reduced to zero; therefore, no connection is lost, i.e., all connections are sent through a switching node.

Recently, two-stage switching networks with *m*-slot connections have been considered in [14,15]. The author considers the SNB, RNB, and RPNB networks. This paper considers the two-stage switching network, which is WNB. Unlike SNB networks, where any new connection will never be blocked, WNB networks always provide a connection path for any future request, provided that an appropriate routing algorithm is used. We propose a routing algorithm and prove the WNB conditions. This routing algorithm establishes fixed assignments between the input and interstage links. This fixed assignment reduces the number of slots in interstage links. As a result, the WNB conditions are the same as the RNB conditions proposed in [15], but WNB networks do not require complicated and time-consuming rearrangements.

The remainder of the paper is organized as follows. Essential related works are summarized in Section 2. In Section 3, we present the two-stage switching network architecture and introduce the notation used in the paper. Section 4 contains the description of the routing algorithm. We also derive WNB conditions in Section 5. In turn, in Section 6, we show some numerical results and comparisons with the SNB and RNB architectures, followed by conclusions.

## 2. Related Work and Contribution

Time-division switching networks have been known since the late 1970s with the appearance of digital time-space switches [16], although Time-Space-Time (TST) switching networks were already considered earlier. Higher capacity switching networks were constructed from integrated switches, and their combinatorial properties, including multirate connections, were considered in many articles, for example, [8,9,10,17,18,19] and summarized in books [1,13]. Clos network variants are now considered in multi-tier data center networks [20]. Four structures of flexible optical switching networks with spectrum converters were proposed in [21]. In [7], two three-stage switching network structures were proposed, named WSW1 and WSW2, which enabled spectrum conversion in the first and third stages, and in [22]—SWS1 and SWS2 switching networks with spectrum conversion capability only in the middle stage. Initially, strict-sense nonblocking conditions for these switching networks were considered, but then rearrangeability conditions and rearrangement algorithms were also dealt with [11,23,24,25]. Studies have shown that the use of the routing algorithm with the functional division of slots in interstage links leads to a significant reduction in the number of switches in the middle section (WSW2 switching networks) or the required number of slots in the interstage links (WSW1 switching networks) [26]. RNB networks [12] are now used primarily in a simultaneous connection model in packet routers, where incoming packets arrive at all switch inputs simultaneously and must be switched to conflict-free output ports. In [15], the use of two-stage switching networks with spectrum converters in each stage and the conditions of SNB, RNB and RPNB were determined. Nonblocking characteristics of elastic optical switches with multicast connections were considered in [27,28], while simulation evaluation of such networks can be found in [29,30,31]. The application of various three-stage elastic optical switches in data center network architectures and their combinatorial properties was proposed in [32,33]. The WNB three-stage switching networks with spectrum converting capabilities in the first and third stages were considered, for example, in [26,34]. The routing algorithm is based on the functional decomposition of the available spectrum on interstage links or center-stage switches. The analysis presented in [26] showed that the lowest complexity of the switching fabric, according to the required equipment, is obtained when the decomposition is carried out in three or four functional sets.

In the case of the two-stage switching fabric, the SNB, RNB, and RPNB have recently been derived in [15]. In this paper, we extend these results and propose the wide-sense nonblocking conditions. In general, the hardware complexity of the WNB switching fabrics is between the SNB (most complex) and the RNB (least complex) one. In the solution shown in this article, the WNB switching network has the same complexity as the RNB one. With more interstage links, it may even contain fewer spectrum converters (which are the most expensive component of a switching network) than the RNB one.

The essential novelties and contributions proposed in this paper can be summarized as follows:

- The proposition of a new algorithm for routing connections in a two-stage switching fabric using the fixed assignment of slots between the input and interstage links of the first-stage switches;
- Derivation of the required number of interstage links and available slots to ensure that each new connection is established using the proposed algorithm, that is, determining wide-sense nonblocking conditions;
- The proposition of the switching network implementation in which the first stage contains only space switches (without spectrum converters) and reduces the number of required spectrum converters by half, compared to rearrangeable and strict-sense nonblocking switching networks.

Traditionally, in algorithms with a functional decomposition of slots, the decomposition into sets is fixed, and within such a set, switches are assigned to connections dynamically. In this proposition, we used a fixed assignment of slots to connections. To the best of our knowledge, this approach has not been addressed in other research. It is also the first time, to the knowledge of the authors, that the WNB and RNB networks reach a similar hardware complexity and, in some cases, WNB can require even less hardware.

## 3. Switching Fabric and Problem Statement

**Figure 1:**
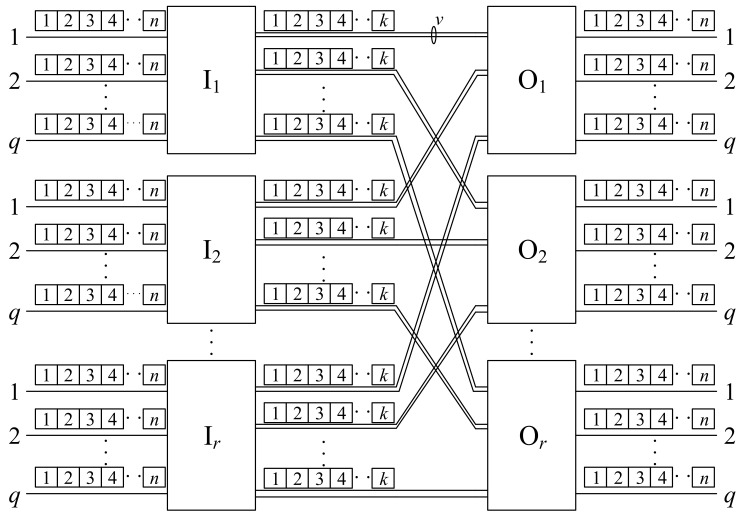
An CC(q,r,v,n,k) switching network.

We consider the two-stage switching network. Switches are arranged in two stages: the input stage and the output stage. Each stage contains *r* switches (generally, these numbers can be different and then the network is asymmetric), denoted by $I_{i}$ and $O_{j}$ for input and output switches, respectively, where 1⩽i,j⩽r. These switches are connected through interstage links. Each $I_{i}$ has *q* input links and vr output links, and *v* links are used to connect to each output switch. Each $O_{j}$ has vr input links and *q* output links. Each link capacity is divided into allocation units, which will be referred to as slots in the rest of the paper; each input and output link has *n* slots, and each interstage link has *k* slots. The two-stage Converting-Converting (CC) switching network is shown in Figure 1. The parameters *q*, *r*, *v*, *n*, and *k* unambiguously define this switching network, and we will denote it by CC(q,r,v,n,k) (by analogy to the three-stage Converting-Space-Converting (CSC) switching networks considered in [26]).

The switching network serves connections of different sizes. The number of slots assigned to one request is indicated by *m*. This value cannot exceed a maximum value $m_{\max}$, that is, $1⩽m⩽m_{\max}⩽n$. We also assume that any *m*-slot connection occupies *m* adjacent slots. This adjacency constraint is imposed in Elastic Optical Networks (EONs) but is sometimes also needed in time-division switching networks [3,26].

A new *m*-slot connection request arrives at the input switch $I_{i}$ and must be set up to the requested output switch $O_{j}$. This connection will be denoted by $\langle I_{i};O_{j};m\rangle$. Because the information on the input/output links and the indexes of the assigned slots are important, we use $\langle I_{i};a;x;O_{j};b;y;m\rangle$ to denote a connection from the input link *a* of $I_{i}$ in slots form *x* to x+m−1, to the output link *b* of $O_{j}$, where the assigned slots range from *y* to y+m−1.

When a new request $\langle I_{i};O_{j};m\rangle$ arrives at the switching node, a routing algorithm selects *m* adjacent and free slots on the interstage link from $I_{i}$ to $O_{j}$. The role of $I_{i}$ is to move information from the slots used in the input link to those used in the interstage link. Similarly, the role of $O_{j}$ is to convert the slots between the interstage and the output links. All switches must have this slot conversion capability; therefore, they are called Conversion Switches (CSs). Since both stages convert slots, the considered structure is the CC switching network.

**Figure 2:**
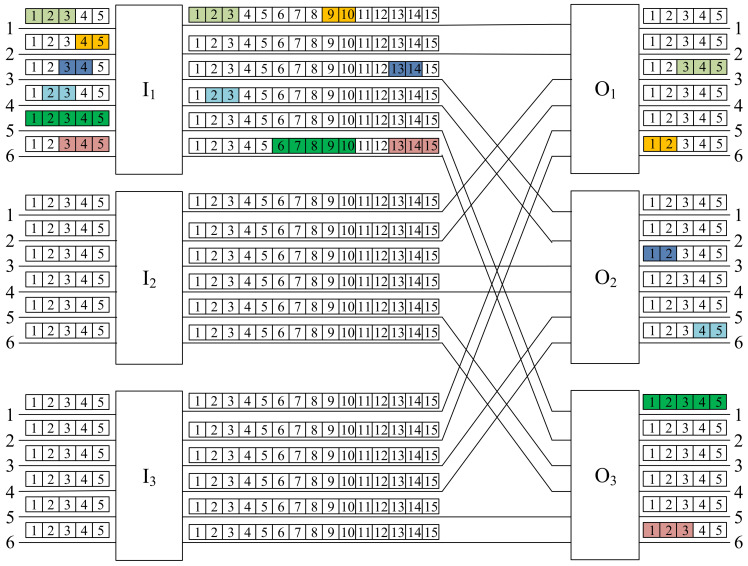
An CC(6,3,2,5,15) switching network.

The example of the CC(6,3,2,5,15) switching network is shown in Figure 2. We have also shown, using different colors, six connections: three 2-slot connections, two 3-slot connections, and one 5-slot connection. According to the proposed notation, these are connections: $\langle I_{1};1;1;O_{1};3;3;3\rangle$, $\langle I_{1};2;4;O_{1};6;1;2\rangle$, $\langle I_{1};3;3;O_{2};3;1;2\rangle$, $\langle I_{1};4;2;O_{2};6;4;2\rangle$, $\langle I_{1};5;1;O_{3};1;1;5\rangle$, and $\langle I_{1};6;3;O_{3};6;1;3\rangle$. As an example, consider the 2-slot connection $\langle I_{1};2;4;O_{1};6;1;2\rangle$. In the input link 2 of the switch $I_{1}$, this connection uses slot numbers 4 and 5. $I_{1}$ moves this connection to its output link 1, connected to $O_{1}$, and converts the slots from 4−5 into 9−10. The role of $O_{1}$ is to direct the connection from its input link 1 to the output link 6 and to convert it from slots 9−10 to 1−2. As seen in this example, the switching network serves connections of different rates (number of slots). In the next section, we consider the routing algorithm, which will be used to establish new requests.

## 4. Routing Algorithm

When a new request arrives at the switching node, the role of a routing algorithm is to assign slots inside an interstage link for the connection. Generally, an algorithm may choose any of the available slots. In the SNB switching network, the new request can always be set up, regardless of the routing algorithm used. The SNB conditions of the CC(q,r,1,n,k) switching networks were considered by Lin in [15]. However, such networks require many slots in the interstage links. Therefore, we propose a new routing algorithm that significantly reduces this number. For further consideration, we assume that *k* is a multiple of *n*, that is, k=αn and α is an integer. In the following description, we will denote slots in links in the following way:

- $s(I_{i},a,x)$—slot *x* in the input link *a* of switch I${}_{i}$;
- $s(O_{j},b,y)$—slot *y* in the output link *b* of switch O${}_{j}$;
- $s(I_{i}O_{j},d,z)$—slot *z* in the interstage link *d* from switch $I_{i}$ to switch $O_{j}$.

**Figure 3:**
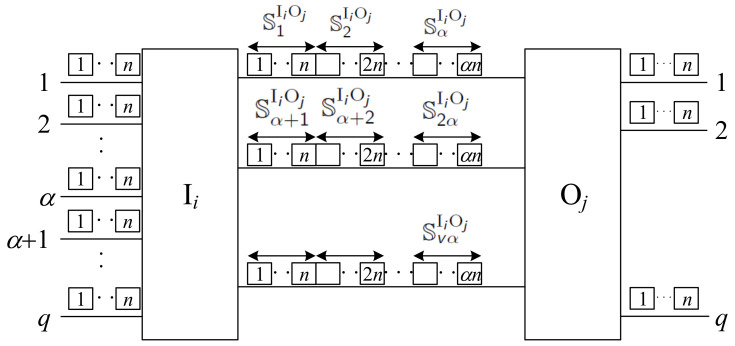
Division of slots in interstage links between switches I${}_{i}$ and O${}_{j}$ into sets.

The proposed algorithm is based on the division of slots in the interstage links into sets, each set containing *n* slots. Between switches $I_{i}$ and $O_{j}$, we have *v* links, so we can create vα sets denoted by $S_{p}^{I_{i}O_{j}}$, where 1≤p≤vα and

(1) $$S_{p}^{I_{i}O_{j}}=\{s\left(I_{i}O_{j},\left\lceil \frac{p}{\alpha }\right\rceil ,n\left((p\;\mathrm{mod}\;\alpha )-1)+1\right);\ldots ;s\left(I_{i}O_{j},\left\lceil \frac{p}{\alpha }\right\rceil ,n\left(p\;\mathrm{mod}\;\alpha \right)\right)\right\},$$

where pmodα denotes the remainder of the division of *p* by α. Thus, we have

(2) $$S_{1}^{I_{i}O_{j}}=\left\{s\left(I_{i}O_{j},1,1\right);\ldots ;s\left(I_{i}O_{j},1,n\right)\right\},$$

(3) $$S_{2}^{I_{i}O_{j}}=\left\{s\left(I_{i}O_{j},1,n+1\right);\ldots ;s\left(I_{i}O_{j},1,2n\right)\right\},$$

etc. This division of slots between sets is shown in Figure 3. We have α sets in one interstage link, each assigned to one input link. When vα>q, vα−q sets on the *v*-th link remain unassigned. Connections from the input link *a* in $I_{i}$ to $O_{j}$ will always use the respective slots in $S_{a}^{I_{i}O_{j}}$. In this way, we have fixed slot assignments between input and interstage links.

**Figure 4:**
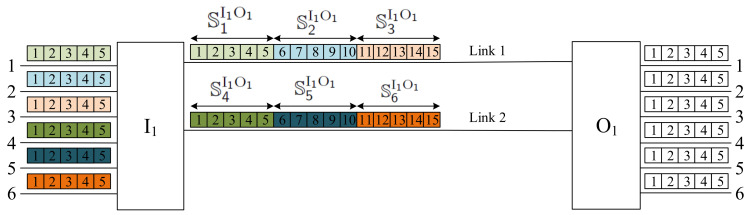
Division of slots in two interstage links between switches $I_{1}$ and $O_{1}$ into sets in case of the switching network presented in Figure 2.

When we consider the switching fabric presented in Figure 2, the division of slots into sets in the interstage links between switches $I_{1}$ and $O_{1}$ is shown in Figure 4. We have two interstage links between switches; each link has k=15 slots. In each input link, we have n=5 slots; that is, the slots in each interstage link should be divided into α=k/n=3 windows. The fixed assignment between the input links and the interstage link slots is as follows (see Figure 4):

- Window 1:   $S_{1}^{I_{1}O_{1}}=\left\{s\left(I_{1}O_{1},1,1\right);\ldots ;s\left(I_{1}O_{1},1,5\right)\right\}$;
- Window 2:   $S_{2}^{I_{1}O_{1}}=\left\{s\left(I_{1}O_{1},1,6\right);\ldots ;s\left(I_{1}O_{1},1,10\right)\right\}$;
- Window 3:   $S_{3}^{I_{1}O_{1}}=\left\{s\left(I_{1}O_{1},1,11\right);\ldots ;s\left(I_{1}O_{1},1,15\right)\right\}$;
- Window 4:   $S_{4}^{I_{1}O_{1}}=\left\{s\left(I_{1}O_{1},2,1\right);\ldots ;s\left(I_{1}O_{1},2,5\right)\right\}$;
- Window 5:   $S_{5}^{I_{1}O_{1}}=\left\{s\left(I_{1}O_{1},2,6\right);\ldots ;s\left(I_{1}O_{1},2,10\right)\right\}$;
- Window 6:   $S_{6}^{I_{1}O_{1}}=\left\{s\left(I_{1}O_{1},2,11\right);\ldots ;s\left(I_{1}O_{1},2,15\right)\right\}$.

Any connection reaching the input link *x* will be established through the slots in the Window *x*. The connections presented in Figure 2 follow this rule.

Let $\langle I_{i};a;x;O_{j};b;y;m\rangle$ be a new valid request, that is, the slots from *x* to x+m−1 on the input link *a* of the switch $I_{i}$, and the slots from *y* to y+m−1 on the input link *b* of the switch $O_{j}$, are free. To assign slots in an interstage link, we propose the algorithm named Fixed Input–interstage Slot Assignment (FISA) presented in Algorithm 1.
**Algorithm 1:** FISA (Fixed Input–interstage Slot Assignment)**Data**: New *m*-slot connection $\langle I_{i};a;x;O_{j};b;y;m\rangle$**Result**: Slots assigned to the considered connection in an interstage link**Assign:****1**    $s\left(I_{i},a,x\right)\to s\left(I_{i}O_{j},\left\lceil \frac{a}{\alpha }\right\rceil ,n\left(a\;\mathrm{mod}\;\alpha \right)-1\right)+x$**2**    $s\left(I_{i},a,x+1\right)\to s\left(I_{i}O_{j},\left\lceil \frac{a}{\alpha }\right\rceil ,n\left(a\;\mathrm{mod}\;\alpha \right)-1\right)+x+1$**3**    ⋮**4**    $s\left(I_{i},a,x+m-1\right)\to s\left(I_{i}O_{j},\left\lceil \frac{a}{\alpha }\right\rceil ,n\left(a\;\mathrm{mod}\;\alpha \right)-1\right)+x+m-1$

**Figure 5:**
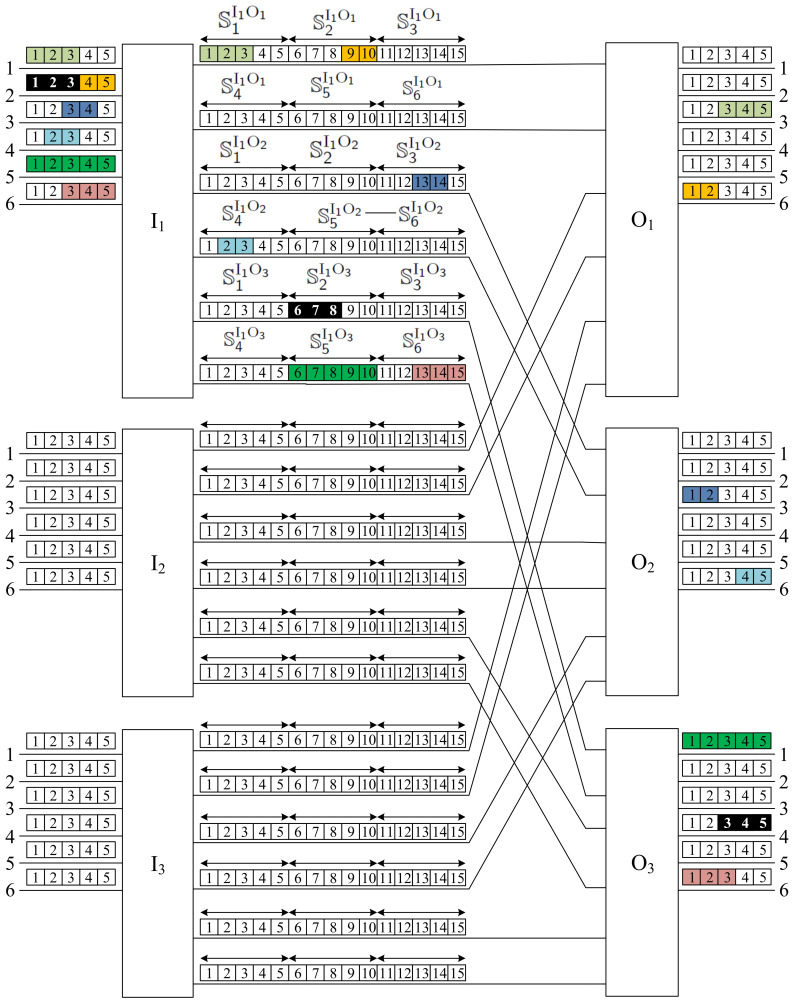
A new request $\langle I_{1};2;1;O_{3};4;3;3\rangle$ (marked in black color) in the CC(6,3,2,5,15) switching network.

We show the operation of the FISA algorithm on the CC(6,3,2,5,15) switching network, where a new request is $\langle I_{1};2;1;O_{3};4;3;3\rangle$. It is presented in Figure 5, and the new request is marked in black. Since this new request is in the input link number 2 of the input switch $I_{1}$, it should be set through $S_{2}^{I_{1}O_{3}}$, which is Window 2 on the interstage link leading to the output switch $O_{3}$. According to the FISA algorithm, the assignment of slots is as follows:

- $s(I_{1},2,1)$→$s(I_{1}O_{3},1,6)$;
- $s(I_{1},2,2)$→$s(I_{1}O_{3},1,7)$;
- $s(I_{1},2,3)$→$s(I_{1}O_{3},1,8)$.

As a result, the connection uses slots 1–3 on link number 2 of input switch $I_{1}$, then moves to slots 6–8 of the first link leading from input switch $I_{1}$ to output switch $O_{3}$, and finally, in switch $O_{3}$, this connection moves to slots 3–5 on output link number 4.

## 5. WNB Conditions

For a new incoming connection $\langle I_{i};a;x;O_{j};b;y;m\rangle$ arriving in the system, the routing algorithm must select one of the *v* links that connect the switches $I_{i}$ and $O_{j}$, and assign *m* adjacent slots inside that link. The input and output switches are SNB-type switches and can perform any slot assignment between its input and output links. It should be noted that SNB-type switches do not ensure that the entire switching network built from such switches is nonblocking. The number of slots *k* in the interstage links for the SNB, RNB, and RPNB operations of the CC(q,r,v,n,k) when v=1 was derived and proved in [15]. Now, we consider the FISA algorithm and show how many *v* and *k* we need to route any request successfully, that is, when this switching fabric is WNB under this algorithm.

**Theorem** **1.** **[FISA]** *The CC(q,r,v,n,k) switching network is WNB according to the FISA algorithm for m-slot connections, where $1⩽m⩽m_{\max}⩽n$, if and only if:*

(4) vk⩾qn.

**Proof** **of** **Theorem 1.** We prove the necessary and sufficient conditions separately.

The necessity is obvious. We have nq input slots on the switch $I_{i}$, and in the “worst-case” scenario, all slots are used by connections to the switch $O_{j}$. To realize all these connections simultaneously, we need at least nq slots in the interstage links.

To prove sufficiency, we show that any connection can always be setup using the FISA algorithm, while all previous connections were also set using this algorithm. The FISA algorithm uses fixed slot assignment, and the slots in each interstage link are divided into windows, where each window contains *n* successive slots. We can formulate $\alpha =\frac{k}{n}$ windows in one link. Since one window is assigned to one input link, we need at least *q* windows in the interstage link. Thus, we need vα⩾q, that is, $v\frac{k}{n}⩾q$ therefore:

(5) vk⩾qn.

The FISA algorithm ensures that, when the slot *x* in the link *a* of the switch $I_{i}$ is free, then the same slot in all sets $S_{a}^{I_{i}-}$ in interstage links from the switch $I_{i}$ to all output stage switches is also free. Therefore, when a new connection $\langle I_{i};a;x;O_{j};b;y;m\rangle$ is valid, the slots from *x* to x+m−1 on the input link *a* are free, and the respective slots in the window $S_{a}^{I_{i}O_{j}}$ are also free and can be assigned to the considered connection. Since switch $O_{j}$ is the SNB-type, the connection from any interstage link that comes to this switch can always be set up to any of its output links. □

An example is shown in Figure 5. We have q=6 input links with n=5 slots for each input switch, k=15 slots in each interstage link, and there are v=2 simultaneous links between each pair of input–output switches $I_{i}$ and $O_{j}$. The WNB conditions are met, since vk=30=qn. In each set of two links between switches $I_{i}$ and $O_{j}$, we exactly have six windows denoted by $S_{1}^{I_{i}O_{j}}$ to $S_{6}^{I_{i}O_{j}}$ (windows in links from switch $I_{1}$ are marked in Figure 5). Six connections marked in different colors are present in the input links of the switch $I_{1}$, and use the respective slots in the appropriate windows as follows:

- $\langle I_{1};1;1;O_{1};3;3;3\rangle ⟶S_{1}^{I_{1}O_{1}}$ slots $s\left(I_{1}O_{1},1,1\right)\to s\left(I_{1}O_{1},1,3\right)$;
- $\langle I_{1};2;4;O_{1};6;1;2\rangle ⟶S_{2}^{I_{1}O_{1}}$ slots $s\left(I_{1}O_{1},1,9\right)\to s\left(I_{1}O_{1},1,10\right)$;
- $\langle I_{1};3;3;O_{2};3;1;2\rangle ⟶S_{3}^{I_{1}O_{2}}$ slots $s\left(I_{1}O_{2},1,13\right)\to s\left(I_{1}O_{2},1,14\right)$;
- $\langle I_{1};4;2;O_{2};6;4;2\rangle ⟶S_{4}^{I_{1}O_{2}}$ slots $s\left(I_{1}O_{2},2,2\right)\to s\left(I_{1}O_{2},2,3\right)$;
- $\langle I_{1};5;1;O_{3};1;1;5\rangle ⟶S_{5}^{I_{1}O_{3}}$ slots $s\left(I_{1}O_{3},2,6\right)\to s\left(I_{1}O_{3},2,10\right)$;
- $\langle I_{1};6;3;O_{3};6;1;3\rangle ⟶S_{6}^{I_{1}O_{3}}$ slots $s\left(I_{1}O_{3},2,13\right)\to s\left(I_{1}O_{3},2,15\right)$.

The new connection $\langle I_{1};2;1;O_{3};4;3;3\rangle$ comes from the 2-nd link of switch $I_{1}$ in slots 1–3; therefore, it will use the slots 6–8 in the window $S_{2}^{I_{1}O_{3}}$. We can see that any window $S_{2}^{I_{1}O_{j}}$, where 1⩽j⩽3 has these slots available for the new connection.

## 6. Comparisons

Now, we compare the results obtained with those presented in [15], and to make the results comparable, we assume v=1. The derived conditions are given in Table 1. Since the various nonblocking conditions do not depend on $m_{\max}$, for SNB, we assumed $m_{\max}=n$. As can be seen, WNB, RNB, and RPNB switching networks require the same number of slots in the interstage links, while, for SNB, this number is much higher. The advantage of WNB networks over RNB and RPNB is that they do not need rearrangements, which are time-consuming and cause a problem with possible interruptions of the connection for a short time. Furthermore, the proposed algorithm is very simplestraightforward with complexity O(1).

**Figure 6:**
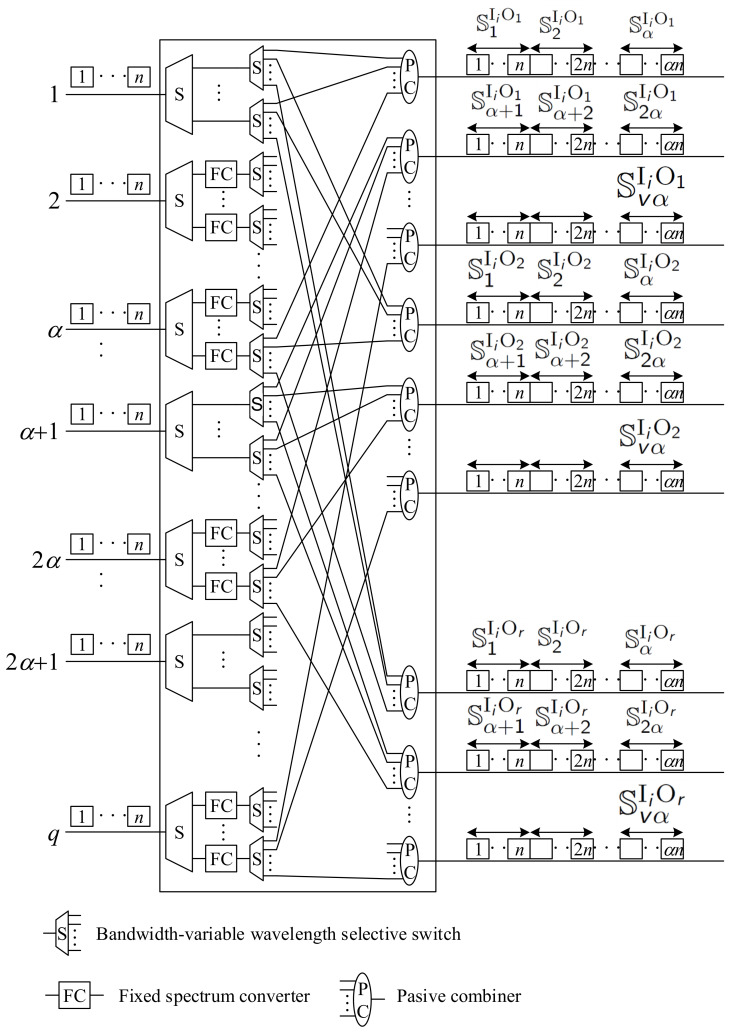
The $I_{i}$ switch with a reduced number of Fixed spectrum Converters (FCs).

The number of slots in interstage links is the same in RNB, RPNB, and WNB switching networks. However, a crucial parameter that affects the cost of the switching network is the number of converters. In EONs, spectrum converters are needed to move user data from one set of frequency slots to another. The architecture of the spectrum conversion switches was presented, for example, in [34,35]. Spectrum converters significantly influence the cost of the switching network. Therefore, to compare optical switching networks, we focus on the number of converters. To ensure the conversion of each slot, the number of converters should be at least the number of slots served by a switch; that is, we need nq converters in each spectrum conversion switch. In the switching networks considered in [15], both stages contain spectrum conversion switches with full-range conversion capability. The required number of Tunable spectrum Converters (TCs) is $C_{\mathrm{TC}}=2rnq$. In the proposed architecture, we can replace the full-range TCs in the first stage with Fixed spectrum Converters (FCs). Furthermore, for each window $S_{x\alpha +1}^{I_{i}O_{j}}$, where 0≤x≤v−1, we do not need this conversion, since the connections in the respective input links use the same slots in the interstage links. In this case, we need only $C_{\mathrm{TC}}^{{}^{\prime }}=rnq$ tunable converters and $C_{\mathrm{FC}}^{{}^{\prime }}=rn\left(q-v\right)$ fixed converters. The internal structure of the input switch $I_{i}$ is shown in Figure 6. Connections are separated into different outputs of the first bandwidth-variable wavelength Selective switch (S) of size 1×n to the converters when conversion is needed. After conversion, they are switched by bandwidth-variable wavelength selective switches of capacity 1×r to appropriate Passive Combiners (PCs), which combine connections from different input links into some interstage links.

By changing the values of *v* and *k*, we can influence the required number of FCs. When we have v=1, we need k⩾qn. When we increase the number of links between stages to two (v=2), the number of slots is reduced to the following:

(6) $$k⩾\left\lceil \frac{qn}{2}\right\rceil .$$

**Figure 7:**
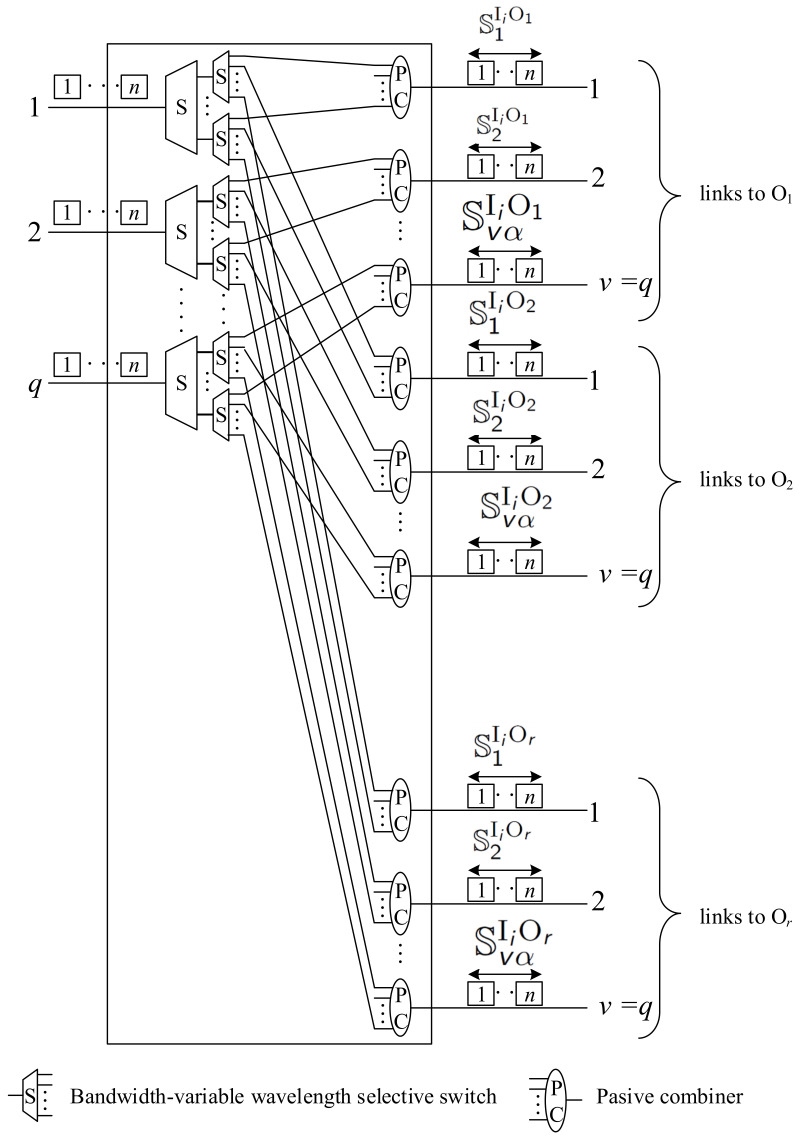
The internal structure of the $I_{i}$ switch when v=q—no converters are needed.

Finally, when v=q, we need k⩾n, and we do not need any spectrum converters in the first stage switches. The internal structure of this switch is shown in Figure 7, and the formulas for calculating the number of spectrum converters are provided in Table 2. In the 2-stage switching networks proposed in [15], as well as in the 3-stage WSW1 and WSW2 switching networks proposed in [7], we need at least 2rqn TCs and none FCs. The FISA algorithm and the WNB conditions of the 2-stage wide-sense nonblocking switching networks considered in this article result in a reduction in the number of TCs by half, to rnq.

## 7. Conclusions

In this paper, we proposed the Fixed Input–interstage Slot Assignment (FISA) algorithm to route *m*-slot connections in two-stage Converting-Converting (CC) switching networks. We derived and proved the Wide-sense Nonblocking (WNB) conditions for this algorithm and have shown that the complexity of the switching network (in terms of the number of slots in interstage links) is the same as for Rearrangeable Nonblocking (RNB) or RePackable Nonblocking (RPNB) switching networks considered in [15]. Furthermore, when used in Elastic Optical Networks (EONs), our solution allows us to implement this switching network with a lower number of Tunable spectrum Converters (TCs), replace part of them with Fixed spectrum Converters (FCs), and even omit some or all of them.

## Figures and Tables

**Table 1:** Nonblocking conditions for the CC(q,r,1,n,k) switching fabrics with $m_{\max}=n$.

SNB	RNB	RPNB	WNB
[15]	[15]	[15]	This Paper
$k⩾\frac{{\left(nq+1\right)}^{2}}{4}$	k⩾nq	k⩾nq	k⩾nq

**Table 2:** The number of TCs and FCs in various CC(q,r,v,n,k) switching fabrics with $m_{\max}=n$.

	SNB/RNB/RPNB	SNB WSW1	SNB WSW2	WNB
	2-Stage	3-Stage	3-Stage	2-Stage
	[15]	[7,34]	[7,34]	This Paper
TCs	2rqn	2rqn	2rqnp	rnq
FCs	0	0	0	rn(q−v)

## Data Availability

Not applicable.

## Abbreviations

The following abbreviations are used in this manuscript:

CC	Converting-Converting
CS	Conversion Switch
CSC	Converting-Space-Converting
DCN	Data Center Network
EON	Elastic Optical Network
FC	Fixed spectrum Converter
FISA	Fixed Input–interstage Slot Assignment
FSU	Frequency Slot Unit
IoT	Internet of Things
PC	Passive Combiner
QoE	Quality of Experience
QoS	Quality of Service
RPNB	RePackable Nonblocking
RNB	Rearrangeable Nonblocking
S	bandwidth-variable wavelength Selective switch
SNB	Strict-sense Nonblocking
TC	Tunable spectrum Converter
TST	Time-Space-Time
WNB	Wide-sense Nonblocking

## References

[1] Kabaciński W. (2005). Nonblocking Electronic and Photonic Switching Fabrics.

[2] ITU-T Recommendation G.694.1 (2012). Spectral Grids for WDM Applications: DWDM Frequency Grid.

[3] Liew S.Y., Wong E.S.K., Fatt C.K. (2010). Advanced information technology of slot-switching network schemes for an all-optical variable-length packet. J. Comp. Sci..

[4] López V., Velasco L. (2016). Elastic Optical Networks. Architectures, Technologies, and Control.

[5] Jinno M., Takara H., Kozicki B., Tsukishima Y., Sone Y., Matsuoka S. (2009). Spectrum-Efficient and Scalable Elastic Optical Path Network: Architecture, Benefits, and Enabling Technologies. IEEE Commun. Mag..

[6] Clos C. (1953). A study of non-blocking switching networks. Bell Syst. Tech. J..

[7] Kabaciński W., Michalski M., Rajewski R. (2016). Strict-Sense Nonblocking W-S-W Node Architectures for Elastic Optical Networks. J. Lightwave Tech..

[8] Turner J.S., Melen R. (2003). Multirate Clos networks. IEEE Commun. Mag..

[9] Liew S., Ng M.-H., Chan C. (1998). Blocking and nonblocking multirate Clos switching networks. IEEE/ACM Trans. Netw..

[10] Jajszczyk A. (2003). Nonblocking, repackable, and rearrangeable Clos networks: Fifty years of the theory evolution. IEEE Commun. Mag..

[11] Lin B.-C. (2016). Rearrangeable W-S-W Elastic Optical Networks Generated by Graph Approaches. IEEE/OSA J. Opt. Commun. Netw..

[12] Beneš V.E. (1965). Mathematical Theory of Connecting Networks and Telephone Traffic.

[13] Hwang F.K. (2004). The Mathematical Theory of Nonblocking Switching Networks.

[14] Lin B.-C. (2019). A New Upper Bound for a Rearrangeable Nonblocking WSW Architecture. IET Commun..

[15] Lin B.C. (2018). Nonblocking Multirate 2-Stage Networks. IEEE Commun. Lett..

[16] Charransol P., Hauri J., Athènes C., Hardy D. (1979). Development of a Time-Division Switching Network Usable in a Very Large Range of Capacities. IEEE Trans. Commun..

[17] Chung S.-P., Ross K.W. (1991). On nonblocking multirate interconnection networks. SIAM J. Comput..

[18] Kabaciński W. (1995). On nonblocking switching networks for multichannel connections. IEEE Trans. Commun..

[19] Kabaciński W., Liotopoulos F.K. (2002). Multirate non-blocking generalized three-stage Clos switching networks. IEEE Trans. Commun..

[20] Singh A., Ong J., Agarwal A., Anderson G., Armistead A., Bannon R., Boving S., Desai G., Felderman B., Germano P. Jupiter Rising: A Decade of Clos Topologies and Centralized Control in Google’s Datacenter Network. Proceedings of the SIGCOMM.

[21] Zhang P., Li J., Guo B., He Y., Chen Z., Wu H. (2013). Comparison of Node Architectures for Elastic Optical Networks with Waveband Conversion. China Commun..

[22] Danilewicz G., Kabaciński W., Rajewski R. (2016). Strict-Sense Nonblocking Space-Wavelength-Space Switching Fabrics for Elastic Optical Network Nodes. J. Opt. Commun. Netw..

[23] Kabaciński W., Al-Tameemie A., Rajewski R. (2019). Rearrangeability of Wavelength-Space-Wavelength Switching Fabric Architecture for Elastic Optical Switches. IEEE Access.

[24] Lin B.-C. (2020). Rearrangeable and Repackable S-W-S Elastic Optical Networks for Connections with Limited Bandwidths. Appl. Sci..

[25] Abuelela E., Żal M. A new control algortithms for simultaneous connection routing in elastic optical networks. Proceedings of the 2021 IEEE 22nd International Conference on High Performance Switching and Routing (HPSR).

[26] Kabaciński W., Abdulsahib M. (2020). Wide-Sense Nonblocking Converting-Space-Converting Switching Node Architecture Under XsVarSWITCH Control Algorithm. IEEE/ACM Trans. Netw..

[27] Danilewicz G. (2019). Asymmetrical Space-Conversion-Space SCS1 Strict-Sense and Wide-Sense Nonblocking Switching Fabrics for Continuous Multislot Connections. IEEE Access.

[28] Danilewicz G. (2019). Supplement to “Asymmetrical Space-Conversion-Space SCS1 Strict-Sense and Wide-Sense Nonblocking Switching Fabrics for Continuous Multislot Connections”—The SCS2 Switching Fabrics Case. IEEE Access.

[29] Sobieraj M., Zwierzykowski P., Leitgeb E. (2021). Determination of Traffic Characteristics of Elastic Optical Networks Nodes with Reservation Mechanisms. Electronics.

[30] Sobieraj M., Zwierzykowski P., Leitgeb E. (2021). Modelling and Optimization of Multi-Service Optical Switching Networks with Threshold Management Mechanisms. Electronics.

[31] Głąbowski M., Ivanov H., Leitgeb E., Sobieraj M., Stasiak M. (2020). Simulation studies of elastic optical networks based on 3-stage Clos switching fabric. Opt. Switch. Netw..

[32] Kabaciński W., Michalski M., Rajewski R., Żal M. Optical Datacenter Networks with Elastic Optical Switches. Proceedings of the IEEE International Conference on Communications (ICC).

[33] Lin B.-C. (2020). Rearrangeable Nonblocking Conditions for Four Elastic Optical Data Center Networks. Appl. Sci..

[34] Kabaciński W., Michalski M., Rajewski R. (2019). Optimization of strict-sense nonblocking wavelength-space-wavelength elastic optical switching fabrics. Opt. Switch. Netw..

[35] Abdulsahib M., Michalski M., Kabaciński W. (2019). Optimization of wide-sense nonblocking elastic optical switches. Opt. Switch. Netw..

